# Adolescent Girls’ Understanding of Tetanus Infection and Prevention: Implications for the Disease Control in Western Nigeria

**DOI:** 10.3389/fpubh.2014.00024

**Published:** 2014-03-27

**Authors:** Adebola Emmanuel Orimadegun, Akinlolu Adedayo Adepoju, Olusegun Olusina Akinyinka

**Affiliations:** ^1^Institute of Child Health, College of Medicine, University of Ibadan, Ibadan, Nigeria; ^2^Department of Pediatrics, College of Medicine, University of Ibadan, Ibadan, Nigeria

**Keywords:** tetanus infection, adolescent girls, tetanus, school-based immunization, toxoid injection, vaccination program, knowledge score

## Abstract

Tetanus is a major cause of morbidity and mortality in developing countries. Nigeria is aiming to eliminate tetanus by maintaining coverage of routine vaccinations for infants and pregnant women, but little attention is given to the adolescents’ needs. This study assessed the understanding of adolescent girls about tetanus infection and prevention in order to provide information that may foster better policy. In this cross-sectional analytical study, 851 female adolescents were selected from eight secondary schools in Ibadan, south-west of Nigeria using a three-stage random sampling technique. A pre-tested structured questionnaire was used to obtain information on demographic and socio-economic characteristics, history of tetanus vaccination, and adolescents’ knowledge of tetanus infection. Mean age of respondents was 14.3 ± 1.9 years. Only 3.1% had received tetanus toxoid injection 1 year prior to the study, most frequently following a “wound or injury” (65.4%). Though 344 (40.4%) respondents claimed that they knew about tetanus as a “serious neurological disease,” only 46.5% correctly defined tetanus. Overall, the mean knowledge score was 4.8 ± 3.1 and 64.7% of the respondents had poor knowledge. Academic class was significantly associated with knowledge; higher mean score among the senior (5.3 ± 5.3) than junior classes (4.4 ± 3.2); *p* < 0.001. Over half (56.2%) of the adolescents disagreed with the statement that “tetanus immunization can be given to students in the school premises.” There is the need to improve immunization campaigns against tetanus among adolescent girls and consider the introduction of school-based immunization programs if the elimination of maternal and neonatal tetanus is to be achieved.

## Introduction

The purpose of this study is to evaluate the understanding of adolescent girls in high school about tetanus and identify factors associated with knowledge of the disease. Tetanus is an acute neurological disease caused by *Clostridium tetani*, a non-invasive, and spore-bearing anaerobic *bacilli* bacterium. *C*. *tetani* infects humans by contaminating wounds, broken skin, or mucous membranes from where it produces the potent toxin, which circulates into the human body to cause the muscle spasms that characterizes the disease (1). Though tetanus affects all ages, it occurs more frequently in children than adults. Tetanus accounted for 20,000–276,000 of neonatal deaths (1% of all child mortality) globally in 2010 (2). In Nigeria, the incidence tetanus ranges from 14.6 to 20 per 1000 live births and it is the leading cause of death in the neonatal age group (3–5). Out of the five million babies born annually in the country, 240,000 (4.8%) die within the first 4 weeks of life and tetanus accounts for up to 20% of these deaths (6). Neonatal tetanus occurs as a result of unhygienic birth practices, most commonly when the spores of *C*. *tetani* contaminate the umbilical cord stump at the time it is cut or dressed after birth (1). Beyond the neonatal age, the portal of entry is usually a contaminated wound or otitis media and it may not be obvious (7).

Tetanus is completely preventable with adequate doses of tetanus toxoid injection (8). The World Health Organisation (WHO) recommends that six doses of tetanus toxoid vaccine be administered before 16 years of age, including three doses in the first year and booster doses at early childhood, school age, and adolescence, respectively. These six doses of tetanus toxoid injections may confer life-long immunity against the disease (8). Using the tetanus toxoid-containing vaccines, concerted efforts toward the global elimination of tetanus commenced since 1989 (9). According to WHO, significant progress has been made since that time, however, as of November 2012, maternal and neonatal tetanus are still major public health problem in 31 countries, including Nigeria (10). Tetanus toxoid vial is distributed regularly by the Nigeria States Ministry of Health and it is meant to be given at no cost to patients who registered at any government health facility. But, a dose of tetanus toxoid may cost about 0.3–1 US Dollar (50–150 naira) in settings like private and mission facilities. It is therefore, difficult to justify the persistence of tetanus in Nigeria despite the availability of cheap, safe, and potent tetanus toxoid vaccine for over 30 years. Though there is an apparent success of the tetanus elimination program in Nigeria, it remains an important endemic infection and the goal of its elimination appears elusive.

The high burden and persistence of tetanus can be viewed against the background of poor uptake of available vaccine among the Nigerian population. This may be partly because of inadequate knowledge of the disease and the consequence of being unprotected. In a recent survey of mothers of children ages 12–23 months Nigeria, Oladokun and colleagues reported that one of the reasons for inadequate vaccination among children was “lack of awareness of the need for additional doses of vaccines” (11). However, data on any association between knowledge and uptake of tetanus vaccine among women of childbearing ages, especially the adolescent girls, are scarce. Available data show that female secondary school enrollments had increased from 33% (12) in 2007 to 43% by 2010 (13) in Nigeria. Thus, high school adolescent girls’ population is substantial and can be targeted with important health information about tetanus. So, it is imperative that they are well informed about the use of preventive measures.

Adequate knowledge regarding tetanus among girls in high schools is even more important not only for personal protection, also for prevention of neonatal tetanus in the future. On the other hand, improper or inadequate knowledge is dangerous and may perpetrate the current poor tetanus toxoid uptake and underutilization of other vaccination programs. Since tetanus vaccine is available at all levels of healthcare, especially in urban areas of Nigeria, poor knowledge and perception of the need to receive it are major likely barriers to receiving it. Since increasing uptake of tetanus vaccine in female adolescents seems an option for improving protection against maternal and neonatal tetanus, there is the need to provide evidence for developing such strategy. If girls in high schools have sufficient and adequate knowledge about tetanus they will pass correct health messages on to the community. Moreover, girls in high schools can readily be reached with five doses of properly spaced tetanus toxoid vaccine, which would most likely provide life-long protection against tetanus in them and their newborns in future (8).

To our knowledge, data on understanding, attitudes and behaviors toward tetanus vaccination among girls in the high schools are scarce in Nigeria. Such information is urgently needed, since the reasons for non-compliance with recommended immunization schedule and the factors that may affect the uptake of other immunizations need to be identified and addressed. It is against this background that the present study was conducted. Its intent was to provide descriptive information that could lead to possible policy and practice interventions.

## Materials and Methods

### Study design and location

This cross-sectional analytical study involved girls in high schools in Ibadan North Local Government Area (IBNLGA), south-west of Nigeria. The IBNLGA has a population of 306,795 (Census 2006) (14), mainly Yoruba-speaking people and comprises urban and slum communities. IBNLGA was purposively selected for this study because the majority of cases of neonatal tetanus seen at the University College Hospital, Ibadan in the 2 years preceding this study resided in the area. There were 28 public and 32 private schools as at the time of the study.

### Study population and sample size calculations

All high school girls in IBNLGA with an estimated population of 25,593 were the target for this study. Those within ages 10–19 years and resident in IBNLGA were eligible to participate but sample size calculation was based on the assumption that 50% of the eligible girls had the correct knowledge regarding tetanus. With a 95% confidence limit and 5% allowable error and by applying the formula for estimating sample size to be selected from an infinite population, 384 high school girls were estimated. However, because of the possibility of clustering of some unknown participants’ characteristics in schools (clusters), a design effect factor of 2 was assumed and the estimated sample size of 384 was multiplied by 2 giving a total of 768 (15). It was also anticipated that some eligible adolescents might decline to participate. Further adjustment was made for a 5% non-response rate; this gave an overall estimated required sample size of 808 participants.

### Sampling method

The participants in the study were essentially a convenience sample, although efforts were made to gain representation from girls of varying ages and different types of schools. A three-stage random sampling technique was used to select eight geo-political wards out of 12 wards, 2 (a private and public each) schools from ward, and 851 students from the list of all selected schools. Eight hundred and sixty-nine were invited, but 18 students whose parents refused to give consent were excluded from the study. Thus, 851 students took part the study. Characteristics of the sample are presented in Table 1. No comprehensive data are available to enable a comparison of participants with the total number of girls attending the sample schools.

**Table 1:** **Demographic characteristics of respondents from in private and public schools**.

Characteristics	All participants (851)		Public schools (710)		Private schools (141)		P*
	*n*	%	*n*	%	*n*	%	
**CLASS**							
Junior (1–3)	410	48.2	59	41.8	351	49.4	0.099
Senior (4–6)	441	51.8	82	58.2	359	50.6	
**ETHNIC GROUPS**							
Yoruba	769	90.4	642	90.4	127	90.1	0.897
Non-Yoruba	82	9.6	68	9.6	14	9.9	
**PARENTS MARITAL STATUS**							
Single/divorced parent	119	14.0	6	4.3	113	15.9	<0.001
Married living together	732	86.0	135	95.7	597	84.1	
**NUMBER OF MOTHER’S CHILDREN**							
1–4	98	11.5	70	9.9	28	19.9	0.001
>4	753	88.5	640	90.1	113	80.1	
**POSITION AMONG MOTHER’S CHILDREN**							
Not first born	606	71.2	94	66.7	512	72.1	0.192
First born	245	28.8	47	33.3	198	27.9	

*p Values for comparison between private and public schools.

### Data collection instrument and procedure

This study was conducted from June to September, 2012. Two visits were made to each of the selected schools: first, to ask the prospective study participants to written informed consent from their parents and request for their immunization record. The purpose of the second visit was to interview students whose parents gave consent and who assented to participate in the study. A structured interviewer-administered questionnaire, pre-tested in high schools at another local government area was used to collect data on age, history of immunization and previous tetanus infection, participants’ knowledge about tetanus, and opinions about giving immunization in school. The investigators were assisted by six trained house physicians.

The time and context for data collection limited the nature and extensiveness of information that could be collected. The instrument focused on the girls’ demographic profile and knowledge. The study was not able to gather contextual information about other factors that might influence either knowledge or health behavior. Knowledge about tetanus was assessed on a 10-point scale, comprising statements and questions (with possible answers as true, false, or I don’t know). “I don’t know” was considered as a wrong answer for all the questions during the analysis and this attracted zero score. A correct answer was awarded a point and a total score of 5–10 was regarded as good knowledge. The questionnaire was reviewed by a three-man panel of experts for content and construct validity. Reliability was established using the pilot test by collecting data from 30 adolescent girls who were not included in the sample. The reliability (internal consistency) of the knowledge scale was tested using the pilot data; the reliability coefficient was 0.859. Adequate explanations of the questions as well as interpretation in local language, as necessary, were made to maximize understanding of the respondents. Much of the data collection was done during the break hour or free periods to avoid disruption of school lessons. The interview lasted for about 15–20 min for each participant.

### Data analysis

Data were analyzed using SPSS for Windows 17.0 (SPSS, Inc., Chicago, IL USA). Participants’ socio-economic class scores were determined based on the occupations and educational attainments of the parents using the classification earlier reported by Oyedeji (16). In this study, a score of 1 point was assigned upper class; 2 and 3 points were grouped as middle class while 4 and 5 points were regarded as lower class. Univariate analysis was done by generating frequencies of the variables, and bivariate analysis was done using Chi-square and Student *t*-test for associations. Socio-economic status, marital status, birth order, and number of children were specifically included in tests of associations because they have been demonstrated to influence immunization rate in previous studies (17, 18). Given the convenience nature of the sample, the descriptive intent of the study and the fact that only two variables (class in school and social class) showed significant association with knowledge at bivariate level of analysis, analyses were limited to univariate methods. All inferential statistics were considered statistically significant if *p* value was less than 0.05.

### Ethical consideration

Approval to carry out the study was obtained from the Oyo State Ministry of Health Research Ethical Review Committee (reference number: AD 13/479/215). Written informed consent was obtained from the participants’ parents or guardians after the purpose of the study was explained to them in writing. A written permission to conduct the study was also obtained from the State Commissioner of Education while the Principals of the selected schools were met to obtain their verbal permission as well. Serial numbers and codes were used to identify participants and schools. The participants were assured that their responses would not be reported individually but as part of an overall study report or scientific publications.

## Results

### Socio-demographic characteristics of the respondents

Study respondents comprised 710 (83.4%) and 141 (16.6%) girls from public and private schools respectively with overall mean age of 14.3 ± 1.9 years. The mean age of respondents from public schools (14.4 ± 1.9 years) was significantly higher than those from private schools (13.6 ± 1.6 years); *p* < 0.001. Other demographic characteristics of the respondents were as shown in Table 1. There were 410 (48.2%) and 441 (51.8%) in junior and senior secondary schools respectively. Most of the participants (90.1% in private and 90.4% in public schools), were of the Yoruba ethnic group while others were non-Yoruba (51 Ibos, 25 Hausas, 4 Urhobo, and 2 Efik). Majority (86.0%) of the respondents were from homes where parents were living together, while few (2.0%) had single parents. Respondents from families with more than four children were 88.5%. Others were from families with four or fewer children. Almost two-thirds (62.4%) of respondents were in the range of second to fourth born of their mothers’ children.

### History of recent immunizations received by the adolescents

Overall, 21.4% of the respondents presented immunization cards with significantly higher number in private school (39.0%) than public schools (17.9%); *p* < 0.001. Only 59 (6.9%) of the respondents had received any form of immunization 1 year prior to the study comprising; 14.9% of those in private schools compared with 5.4% in public schools (*p* < 0.001). The following vaccines were received by the participants: tetanus toxoid (*n* = 26; 3.1%), measles (*n* = 17; 2.0%), polio (*n* = 14; 1.6%), yellow fever (*n* = 13; 1.5%), Hepatitis B (*n* = 9; 1.1%), cerebrospinal meningitis (*n* = 9; 1.1%), MMR (*n* = 17; 2.0%), and pneumococcal (*n* = 13; 1.5%). There were no significant differences in the numbers of respondents who were immunized 1 year prior to the study between private and public schools for most vaccines except tetanus toxoid.

Majority, 17 (65.4%) of the 26 mentioned “wound/injury” as the reason for being given tetanus toxoid injection. Of the 26 participants who received tetanus immunization in the 1-year prior to the study, 11 (42.3%) and 10 (38.5%) were given at primary health centers and private clinics, respectively. The remaining four participants received tetanus toxoid injections at mission health facilities (*n* = 3; 11.5%) and patent medicine stores (*n* = 2; 7.7%).

### Knowledge of the adolescents about tetanus infection and prevention

While 344 (40.4%) respondents claimed that they knew about tetanus as an “acute serious disease,” only 160 (46.5%) of them correctly defined it. Table 2 shows the responses of the adolescents to various questions and statements about tetanus. Most of the respondents (65.5%) said that “tetanus is an infectious disease caused by contamination of wound” while 3.3% said it was not true. Less than half (47.2%) of the respondents marked true for the statement: “the agent responsible for tetanus can be found throughout the environment, usually in soil, dust, and animal waste” and 42.3% did not know. Concerning the statement “tetanus is acquired through contact with the environment; it is not transmitted from person to person,” 19.2% of the respondents chose false while 43.0% did not know. The majority (63.9%) of respondents did not know that tetanus results in severe, uncontrollable muscle spasms. Twenty-three per cent of the respondents agreed that muscle spasms are the cause of the “lockjaw” in people suffering from tetanus. Almost half (46.1%) of the respondents did not know that tetanus may develop in people who were not immunized against it or in people who have failed to maintain adequate immunity with active booster doses of vaccine while 45.1% said it was true. The majority (70.9%) of the respondents marked true for the statement: “if people have a wound, they should seek medical attention.” Almost half (48.5%) of the respondents agreed that “for individuals who were not immunized against tetanus or have not kept up tetanus booster shots every 10 years, any open wound is at risk of developing tetanus” while only 5.6% thought it is false. Fifty-eight percent said it was true that if individuals have trouble swallowing or have spasms in the facial muscles, they should go to the emergency department for treatment immediately. Only 32.7% of respondents said that the statement “any wound that results in a break in the skin should be cleaned with soap and running water in order to prevent tetanus” is true, while 29.3% of respondents were correct having said it is not true. More than half (57.5%) of the respondents agree that people who were not completely immunized and have wounds should receive a tetanus immunization.

**Table 2:** **Responses of the high school girls to various statements on tetanus**.

Statements	True		False		Don’t know	
	*n*	%	*n*	%	*n*	%
Tetanus is an infectious disease caused by contamination of wounds	557^a^	65.5	28	3.3	266	31.2
The agent responsible for tetanus is found throughout the environment, usually in soil, dust, and animal waste	402^a^	47.2	89	10.5	360	42.3
Tetanus is acquired through contact with the environment; it is not transmitted from person to person	322^a^	37.8	163	19.2	366	43.0
Tetanus results in severe, uncontrollable muscle spasms. For example, the jaw is “locked” by muscle spasms, causing the disease to sometimes be called “lockjaw”	201^a^	23.6	106	12.5	544	63.9
Tetanus may develop in people who are not immunized against it or in people who have failed to maintain adequate immunity with active booster doses of vaccine	384^a^	45.1	75	8.8	392	46.1
If people have a wound, they should seek medical attention	603^a^	70.9	19	2.2	229	26.9
If they are not immunized against tetanus or have not kept up tetanus booster shots every 10 years, any open wound is at risk of developing tetanus	413^a^	48.5	48	5.7	390	45.8
If individuals have trouble swallowing or have muscle spasms in the facial muscles, go to the emergency department for treatment immediately	494^a^	58.0	33	3.9	324	38.1
Any wound that results in a break in the skin should be cleaned with soap and running water in order to prevent tetanus	278	32.7	249^a^	29.2	324	38.1
People who are not completely immunized and have wounds should receive a tetanus immunization	489^a^	57.4	39	4.6	323	38.0

*^a^Correct answers to respective statements*.

The overall mean knowledge score was 4.8 ± 3.1. Almost two-thirds (64.7%) of the respondents had poor knowledge. Table 3 shows the comparisons of mean knowledge scores and categories by socio-demographic characteristics of respondents. Of the seven socio-demographic characteristics examined in this study, only academic class of the adolescents was significantly associated with knowledge of the respondents with those in the senior classes having higher knowledge score and fewer respondents in the poor knowledge group than the junior classes.

**Table 3:** **Knowledge scores of the adolescents about tetanus compared by socio-demographic variables**.

Characteristics	Mean knowledge score	*p*	Poor knowledge		Good knowledge		*p*	OR (95% CI)
			*n*	%	*n*	%	
**CLASS IN SCHOOL**								
Junior (1–3)	4.4 ± 3.2	<0.001	290	70.7	120	29.3	<0.001	1.67 (1.25, 2.22)
Senior (4–6)	5.3 ± 2.3		261	59.2	180	40.8		1
**TYPE OF SCHOOL**								
Private	5.2 ± 2.5	0.060	93	66.0	48	34.0	0.742	1.07 (0.73, 1.56
Public	4.8 ± 3.1		458	64.5	252	35.5		1
**ETHNIC GROUPS**								
Yoruba	4.6 ± 3.0	0.531	496	64.5	273	35.5	0.643	0.89 (0.55, 1.45)
Non-Yoruba	4.2 ± 3.2		55	67.1	27	32.9		1
**PARENTS MARITAL STATUS**								
Single/divorced parent	3.8 ± 2.9	0.124	80	67.2	39	32.8	0.542	1.14 (0.75, 1.72)
Married living together	4.9 ± 2.0		471	64.3	261	35.7		
**NUMBER OF MOTHER’S CHILDREN**								
1–4	4.7 ± 2.7	0.581	68	69.4	30	30.6	0.307	1.27 (0.80, 1.99)
>4	4.9 ± 3.1		483	64.1	270	35.9		
**POSITION AMONG MOTHER’S CHILDREN**								
Not first born	4.8 ± 3.1	0.890	385	63.5	221	36.5	0.243	0.83 (0.61, 1.14)
First born	4.9 ± 2.9		166	67.8	79	32.2		
**SOCIAL CLASS**								
Upper	5.0 ± 3.1	0.507	113	59.5	77	40.5	–	1
Middle	4.7 ± 3.0		228	67.5	110	32.5	0.066	1.41 (0.97, 2.04)
Lower	4.8 ± 3.1		210	65.0	113	35.0	0.210	1.27 (0.88, 1.83)

*OR, odds ratio; CI, confidence interval*.

### Opinion of the adolescents about tetanus vaccination in the school premises

The distributions of participants’ responses to all the six opinion statements were not significantly different between those who had poor and good knowledge. The responses of the respondents on whether they disagreed, agreed, or they were indifferent to a list of the six opinion statements on tetanus vaccination within the school premises were as shown in Table 4. Over half (56.2%) of respondents disagreed with the statement that “tetanus immunization can be given to students in the school.” Similarly, about half of the respondents agreed with the statements: “parents will not agree that I should be given injection in school even if they know the benefits” (50.2%) and “it is not possible to give immunization in the form of injections in school premises” (48.8%).

**Table 4:** **Opinions of high school girls about tetanus vaccination in the school premises**.

	Disagreed		Agreed		Indifferent	
	*n*	%	*n*	%	*n*	%
Tetanus immunization can be given to students in the school	478	56.2	306	36.0	67	7.9
My parents will not agree that I should be given injection in school even if they know the benefits	377	44.3	427	50.2	47	5.5
It is not possible to give immunization in the form of injections in school premises	353	41.5	415	48.8	83	9.8
I think majority of students will receive tetanus immunization in school if government made it compulsory	462	54.3	317	37.3	72	8.5
Tetanus injection in school will be feasible if nurses were stationed in the schools to administer it	455	53.5	302	11.0	94	11.0
Awareness and knowledge among students and teachers concerning the importance of tetanus toxoid injection is low	377	44.3	328	38.5	146	17.2

Regarding payment for tetanus immunization if introduced into the school health program, majority (79.1%) stated that government should be responsible for funding it. Only 20.9% of all respondents said that parents, government and non-governmental organizations should be responsible for the cost of tetanus vaccination in schools.

## Discussion

The low uptake of health interventions in Nigeria particularly vaccination against tetanus is a cause for concern as the nation remains among 31 countries yet to eliminate maternal and neonatal tetanus in spite of the global efforts at eradicating the disease (10). Among the most neglected population, but with the potential for passing the right information about tetanus to the Nigerian communities, are the adolescent girls in schools. If this group of individuals could be reached with correct information and given access to vaccination in school as it is the case in countries like Malaysia, Indonesia, Sri Lanka, and Tunisia (19), the goal of eradicating tetanus may be achieved faster than currently imagined. It was on this premise that this study evaluated the understanding of high school girls about tetanus in a local government area in the south-west of Nigeria. The study has revealed that the level of knowledge of tetanus and immunization against the disease is poor among adolescent girls. Over half (56.2%) of the participants disagreed with the statement that “tetanus injection in schools could be feasible even if nurses were stationed in the schools to administer it.” Majority (79.0%) opinion was that government should be made to pay for tetanus vaccination if introduced into the current school health program.

As at 2011, the national Diphtheria–Pertussis–Tetanus vaccine (DPT-3) coverage was 47% and only 60% of women received tetanus toxoid during pregnancy (20). While the record of tetanus toxoid booster doses in late childhood is scarce, the adolescents are not currently catered for in the immunization program. The low uptake of tetanus toxoid-containing vaccine reported in this study (3.1%) suggests the need to consider commencement of vaccination among girls as early as school age. However, despite the high number of girls who had not received tetanus toxoid injection for more than a year before this study, over half of participants disagreed with the statement that “tetanus injection in school could be feasible if nurses were stationed in the schools to administer it.” The implication of this finding is that participants generally lack insight into the magnitude of the problem posed by the disease, the risk associated with tetanus and available means of prevention. For instance, only about 24% were aware of the fact that tetanus may result in severe, uncontrollable muscle spasms and lockjaw. Just a little over half of the participants considered it important that those who are not immunized need to seek medical attention in order to be given tetanus immunization.

The fact that the general knowledge of the adolescent girls about tetanus was poor is not surprising. It has previously been shown that knowledge about tetanus can be that poor even among healthcare workers in countries with high burden of the disease (21). Though tetanus as a disease may be mentioned in some of the subjects that are taught in schools, it is not clear how much of such information students would be able to recall. A similar study, which involved only 114 adolescent girls in rural Haryana, India, showed that though tetanus immunization coverage was better among school going adolescents than those out of school, none of girls could tell the correct immunization schedule for children, indicating poor knowledge as seen in the present study (22).

Overall, the adolescent girls who participated in the present study had negative attitudes toward the introduction of school-based immunization program. It appears from these data that majority would not support the initiative. However, if the Nigerian government decides to introduce tetanus vaccination in school as it was done for smallpox in the early 1970s, the opinion of the majority (73.0%) of respondents was that government should be made to pay for it. Generally, it seems the school girls appreciated neither the seriousness of tetanus infection nor the need to seek protection. The respondents’ position on the issue of school-based immunization could partly be a reflection of the attitude of adolescents to immunization and reflects the enormity of the efforts that must be put in if tetanus immunization is to be successfully incorporated into the current school health program in Nigeria.

School-based programs for administering booster doses of tetanus toxoid may be the best strategy to cover the gap in immunity against tetanus between routine immunization of infancy with three doses of DPT and tetanus toxoid being administered to women of childbearing age. Such school-based program depends on high female school enrollment and low dropout rates for its success. The moderately high levels of school attendance in the urban areas of the south-west of Nigeria (13) makes this strategy workable. In Nigeria, secondary school participation ratio was 43% as at 2010 (13) and may be improving. Therefore, a school health program including tetanus vaccination may be a feasible option for improving coverage among adolescent girls who otherwise may not be vaccinated during pregnancy. Other advantages of immunizing school girls include: the opportunity to raise health awareness at an early age and to encourage the use of personal vaccination records (19, 23). School-based delivery of health interventions is currently of great interest and, in developing countries, could be combined with delivery of other health interventions such as distribution of folate supplementation and vitamin A (23). Though, vaccinating adolescent girls against tetanus could bring about substantial long term gains, its implementation will require an assessment of the proportion of girls who can be reached in schools on vaccination days. Further study to assess the acceptability and cost-effectiveness will be needed for effective policy formulation in Nigeria.

The present study has policy implications and it is relevant to clinical practice for many reasons. The persistence of tetanus as the cause of high proportion of deaths among Nigerian children in spite of availability of vaccines and past efforts made toward immunization against tetanus and the high fatality rate of tetanus underscores the need to assess the level of understand and extent of perceived protection among adolescent population. Also, universal immunization of school adolescents is one of the promising strategies that may reduce the burden of tetanus in Nigeria and these data have provided some insight into some aspects needing attention. It is clear from this study that before policy-makers would apply the school-based approach to the reduction of tetanus burden in Nigeria; there is the need to further assess its acceptability among the children and their caregivers including parents and teachers. Furthermore, there is also the need to consider strategies that would take care of those adolescents who are not in school as well as barriers to receiving vaccinations in the population. These aspects constitute a potential area for future research. Nonetheless, data on knowledge about tetanus immunization among female adolescents are essential for effective implementation of the current WHO tetanus immunization policy. Another relevance of this study is that it has provided some insight into the association between self-reported vaccination history and socio-demographics.

Though this study is the first to provide detailed information about the knowledge, attitudes, and behavior of schooling adolescent girls in Nigeria toward tetanus, the restriction of the participants to one local government area limits the generalizability of the data. Also, that only 21.4% of the respondents could provide immunization cards limits the validity of the information obtained from the research participants on previous immunization uptake. Given the fact that 64.7% of the participants eventually had poor knowledge, a *post hoc* power calculation showed that the study was adequately powered (84%). We are reasonably certain, within the limit of the study power, that the data is representative of the adolescents in the study area. However, it is not known whether responses of adolescents in the schools not selected for the study and those not in schools would have altered the interpretation of these data.

Another important point that needs to be considered while interpreting findings in this paper is that no data were collected on other factors such as accessibility to immunization, availability of vaccines, and health-seeking behaviors of participants and their family members. These factors have been shown to significantly affect immunization rates in Nigeria (24–26). The fact remains that the present data suggest that strengthening vaccination in schools should be considered in tetanus control to achieve the desired high coverage and it could be achieved by identifying populations without access to routine vaccinations such as the adolescent girls and providing sustainable outreach services to such groups. To further strengthen the conclusion of findings in this study, additional analysis using multivariate methods may be undertaken in the future, but the intent of the study reported here was to provide initial descriptive information.

## Conclusion

In all, it is apparent from the data that the understanding of adolescents in study area is less than average and majority may object to the offer of immunization in the school premises. Therefore, there is the need to improve immunization campaign against tetanus among adolescents in high schools in Nigeria. An inclusion of health education focused on tetanus might result in change in adolescents’ disposition as well as increase immunization uptake in the schools. Policy-makers need to consider the introduction of school-based immunization program if the elimination of maternal and neonatal tetanus is to be achieved.

## Author Contributions

Adebola Emmanuel Orimadegun conceptualized and designed the study, analyzed the data and participated in writing the manuscript. Akinlolu Adedayo Adepoju participated in the design of the study, supervised collection of data and contributed to writing the manuscript. Olusegun Olusina Akinyinka contributed to design of the study, interpretation of results and writing of the manuscript. All authors read and approved the final manuscript.

## Conflict of Interest Statement

The authors declare that the research was conducted in the absence of any commercial or financial relationships that could be construed as a potential conflict of interest.
